# Novel small molecular compound 2JY-OBZ4 alleviates AD pathology in cell models via regulating multiple targets

**DOI:** 10.18632/aging.204336

**Published:** 2022-10-12

**Authors:** Qian Guo, Gang Wu, Fang Huang, Zhen Wei, Jian-Zhi Wang, Bin Zhang, Rong Liu, Yang Yang, Xiaochuan Wang, Hong-Lian Li

**Affiliations:** 1 School of Basic Medicine, Key Laboratory of Education Ministry, Hubei Province of China for Neurological Disorders, Tongji Medical College, Huazhong University of Science and Technology, Wuhan 430030, China; 2 Co-innovation Center of Neuroregeneration, Nantong University, Nantong JS 226001, China; 3 Institutes of Biomedical Sciences, School of Medicine, Jianghan University, Wuhan 430056, China; 4 Hubei Key Laboratory of Natural Medicinal Chemistry and Resource Evaluation, School of Pharmacy, Huazhong University of Science and Technology, Wuhan 430030, China; 5 Shenzhen Huazhong University of Science and Technology Research Institute, Shenzhen 518000, China

**Keywords:** Alzheimer’s disease (AD), Huperzine A (Hup-A), beta-secretase 1 (BACE1), protein phosphatase- 2A (PP2A), cholinesterase inhibitor

## Abstract

Alzheimer’s disease (AD) is the most common form of neurodegenerative dementia, characterized by cognitive deficits and memory dysfunction, which is clinically incurable so far. Novel small molecular compound 2JY-OBZ4 is one of structural analogue of Huperzine A (Hup-A), an anti-AD drug in China. In our previous work, 2JY-OBZ4 exhibited potent effects on tau hyperphosphorylation, Aβ production and acetylcholinesterase (AChE) activity. However, 2JY-OBZ4's anti-AD effects and the underlying molecular mechanisms remain unclear. We here reported that 2JY-OBZ4 resisted tau hyperphosphorylation at Thr181 and Ser396 sites in HEK293-hTau cells transfected with GSK-3β, decreased tau phosphorylation via upregulating the activity of PP2A in HEK293-hTau cells and reduced Aβ production through regulating protein levels of APP cleavage enzymes in N2a-hAPP cells. Meanwhile, we found that 2JY-OBZ4 had no adverse effects on cell viability of mice primary neuron even at high concentration, and ameliorated synaptic loss induced by human oligomeric Aβ42. 2JY-OBZ4 had moderate AChE inhibitory activity with the half maximal inhibitory concentration (IC50) to be 39.48 μg/ml *in vitro*, which is more than two times higher than Hup-A. Together, 2JY-OBZ4 showed promising therapeutic effects in AD cell models through regulating multiple targets. The research provides a new candidate for the therapeutic development of AD.

## INTRODUCTION

Alzheimer’s disease (AD) is the most common neurodegenerative disorder characterized clinically by cognitive dysfunctions and progressive memory loss [1]. The increased prevalence of AD not only brings severe and irreversible damage to patients’ life, but also become a financial burden to societies and families [2]. As pathogenesis of AD is still unclear, there is currently no effective therapy for AD, highlighting an urgent need for drug development.

Intracellular accumulation of hyperphosphorylated tau and extracellular aggregation of amyloid beta (Aβ) are pathological hallmarks in AD brains [3], and have been recognized as significant targets for pharmaceutical development of AD [1, 4, 5]. In addition, acetylcholinesterase (AChE) inhibition has been a classic target for AD since 1980s [6, 7]. Nevertheless, in recent years, therapies targeting at tau pathology, Aβ disposition or AChE turned out to be disappointing in clinical study [4, 5, 8]. Given the multifactorial characteristics and underlying complexity of AD pathologies, the expectation that a single agent targeting a single pathological pathway would be highly effective in slowing disease progression seems to be irrational. Hence, therapeutics based on multiple targets would be tendency for the therapeutic development of AD.

Huperzine A (Hup-A), an approved anti-AD drug in China, has been found to act on multiple AD targets, such as inhibiting AChE, inhibiting N-methyl-D-aspartate (NMDA) receptor, protecting neuronal cells against Aβ, free radicals and hypoxia-ischemia induced injury and so on [9–12]. Unfortunately, Hup-A induces annoying cholinergic side effects as nausea, vomiting and diarrhea [13], making the therapy difficult to continue. As we know, there is closely relationship between structure and bioactivity for compounds. Thus, it has been caught attention to search for Hup-A’s structural analogues, in order to find a compound with higher activity and effectiveness. Seven lycophyte compounds, including Lyconadin A, Lyconadin B, Lyconadin C, Lyconadin D, Lyconadin E, H-R-NOB and 2JY-OBZ4, were synthesized chemically [14]. Previous research found that among the seven compounds, 2JY-OBZ4 showed the most potent anti-AD effects like decreasing hyperphosphorylated tau, Aβ production and inhibiting acetylcholinesterase (unpublished data). However, the molecular mechanisms underlying these effects remain unknown. In the present study, as Hup-A’s anti-AD effects have been exhaustively elucidated by previous studies [9–12], we use Hup-A as positive control in the subsequent experiments. We examined the activity of major tau phosphatase and kinase by western blot and enzyme kits. Then the levels of APP cleavage enzymes were detected to illustrate the underlying mechanisms for decreased Aβ production. To quantify 2JY-OBZ4’s inhibitory activity on AChE, AChE activity was evaluated after treatment with gradient concentrations of 2JY-OBZ4 in primary neuron, whereafter, half maximal inhibitory concentration (IC50) was figured out. We found that 2JY-OBZ4 showed promising therapeutic effects in AD cell models through regulating multiple targets mentioned above.

## RESULTS

### Novel small molecular compound 2JY-OBZ4 is a structural analogue of anti-AD drug, Huperzine A

**Figure 1:**
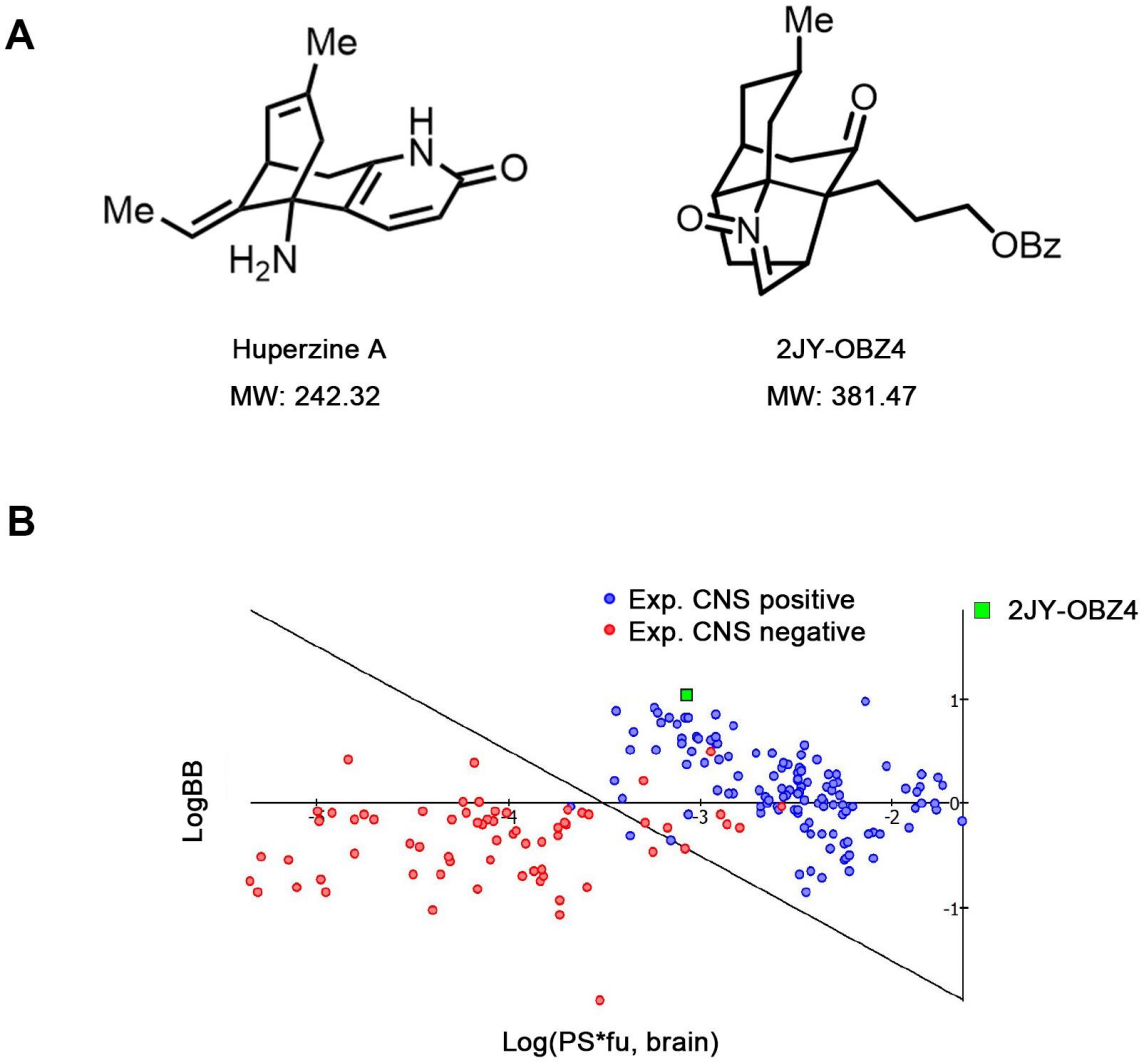
**Novel small molecular compound 2JY-OBZ4 is a structural analogue of anti-AD drug, Huperzine A.** (**A**) Chemical structure of 2JY-OBZ4 and Huperzine A (Hup-A). (**B**) Scatter plot to compare relevant brain penetration characteristics of 2JY-OBZ4 to a set of well-known CNS and peripheral drugs processed by ACD/Percepta. Log(PS*fu, brain) means brain/plasma equilibration rate; LogBB means extent of brain penetration.

2JY-OBZ4 is a structural analogue of Huperzine A (Hup-A), an approved AD drug in China and an approved food supplement in USA [13]. In our previous study, 2JY-OBZ4 was confirmed to have promising anti-AD effects in cell models (unpublished data). 2JY-OBZ4 has a molecular mass of 381.47 dalton, 139 dalton larger than Hup-A (Figure 1A). Structurally, 2JY-OBZ4 possesses a common heterocyclic frame and a common carbonyl oxygen with Hup-A. Structure of carbonyl oxygen has been thought to play a predominant part in the bioactivity of inhibiting AChE [15]. Thus, we thought 2JY-OBZ4’s carbonyl oxygen structure may be the reason for its certain AChE inhibition activity (unpublished data).

For anti-AD drug, the ability to cross the blood-brain barrier (BBB) is crucial. Hup-A was found to penetrate BBB smoothly [16]. To penetrate BBB via lipid-mediated free diffusion, small molecular compound should probably have a molecular weight < 400 Da and forms < 8 hydrogen bonds (represents for high lipid solubility) [17]. Fortunately, 2JY-OBZ4 meets the above criteria. Furthermore, lipid/water partition coefficient LogP and LogD were predicted by using ACD/Percepta software. As a result, both LogP and LogD were 4.62 under different pH conditions, which indicated that 2JY-OBZ4 has strong lipid solubility. In the meanwhile, BBB permeability was predicted *in silico* by the same software, which identified 2JY-OBZ4 as a sufficient compound to penetrate brain for CNS activity (Figure 1B). These findings supported that 2JY-OBZ4 can cross BBB easily.

### 2JY-OBZ4 resisted tau hyperphosphorylation in HEK293-hTau cells transfected with GSK-3β plasmid

In our previous research, 16 μM 2JY-OBZ4 could alleviate AD pathologies, such as decreasing tau phosphorylation and Aβ production (unpublished data). CCK-8 assay showed that various concentrations of 2JY-OBZ4 (250 nM, 500 nM, 1 μM, 2 μM, 4 μM, 8 μM, 16 μM, 32 μM and 64 μM) had no adverse effects on HEK293-hTau cells (unpublished data). Moreover, 1 μM and 8 μM 2JY-OBZ4 increased the cell viability (unpublished data). To further explore the underlying mechanisms, we treated AD cell models with different concentrations of 2JY-OBZ4 (1 μM, 4 μM, 16 μM and 64 μM), while 10 μM Hup-A was served as a positive control in accordance to Wang CY et al.’s research [12].

**Figure 2:**
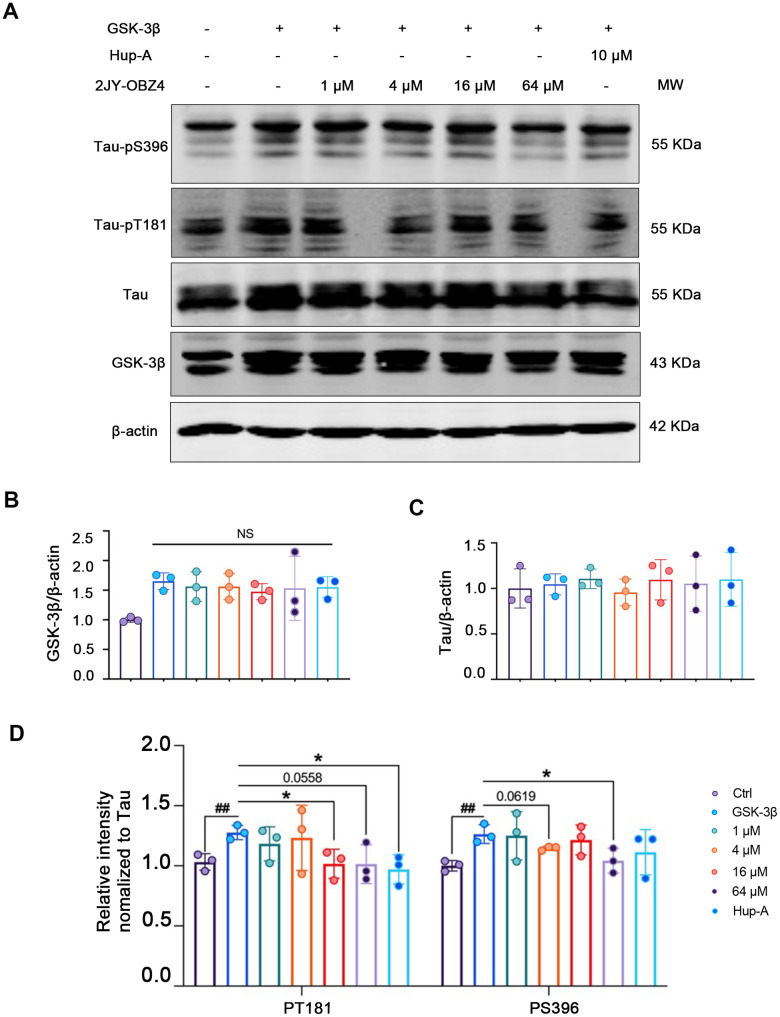
**2JY-OBZ4 resisted tau hyperphosphorylation in HEK293-hTau cells transfected with GSK-3β plasmid.** (**A**) Western blots and (**B**–**D**) quantitative analysis for GSK-3β, Tau, tau-pT181 and tau-pS396 in HEK293-hTau cells overexpressed with GSK-3β. MW Molecular weight. n = 3 per group. NS means no significance, p value significance is calculated from a one-way ANOVA, data are represented as mean ± SEM. *p < 0.05 and ##p < 0.01.

First, we wanted to measure 2JY-OBZ4’s effects on hyperphosphorylation of tau. To enlarge the level of phosphorylated tau in HEK293-hTau cells (with stable hTau expression), we transfected HEK293-hTau cells with glycogen synthase kinase-3β (GSK-3β) plasmid. GSK-3β is the main kinase of tau phosphorylation, which could robustly increase phosphor-tau level [18]. After treatment with various concentrations of 2JY-OBZ4 and 10 μM Hup-A for 24 h, western blot results showed that GSK-3β was increased in all transfected groups, and there was no significant change observed (Figure 2A, 2B). Furthermore, there was no difference on tau level among all groups (Figure 2A, 2C). Interestingly, 16 μM and 64 μM 2JY-OBZ4 decreased tau hyperphosphorylation of Thr181 site; 4 μM and 64 μM 2JY-OBZ4 decreased tau hyperphosphorylation of Ser396 site (Figure 2A, 2D). As positive control, Hup-A decreased tau phosphorylation of Ser396 site (Figure 2A, 2D). To conclude, 2JY-OBZ4 exhibited better effects on resisting tau hyperphosphorylation than Hup-A, in particular, the concentration of 64 μM showed the most potent efficiency.

### 2JY-OBZ4 induced tau dephosphorylation in HEK293/tau cells via upregulating the activity of PP2A

**Figure 3:**
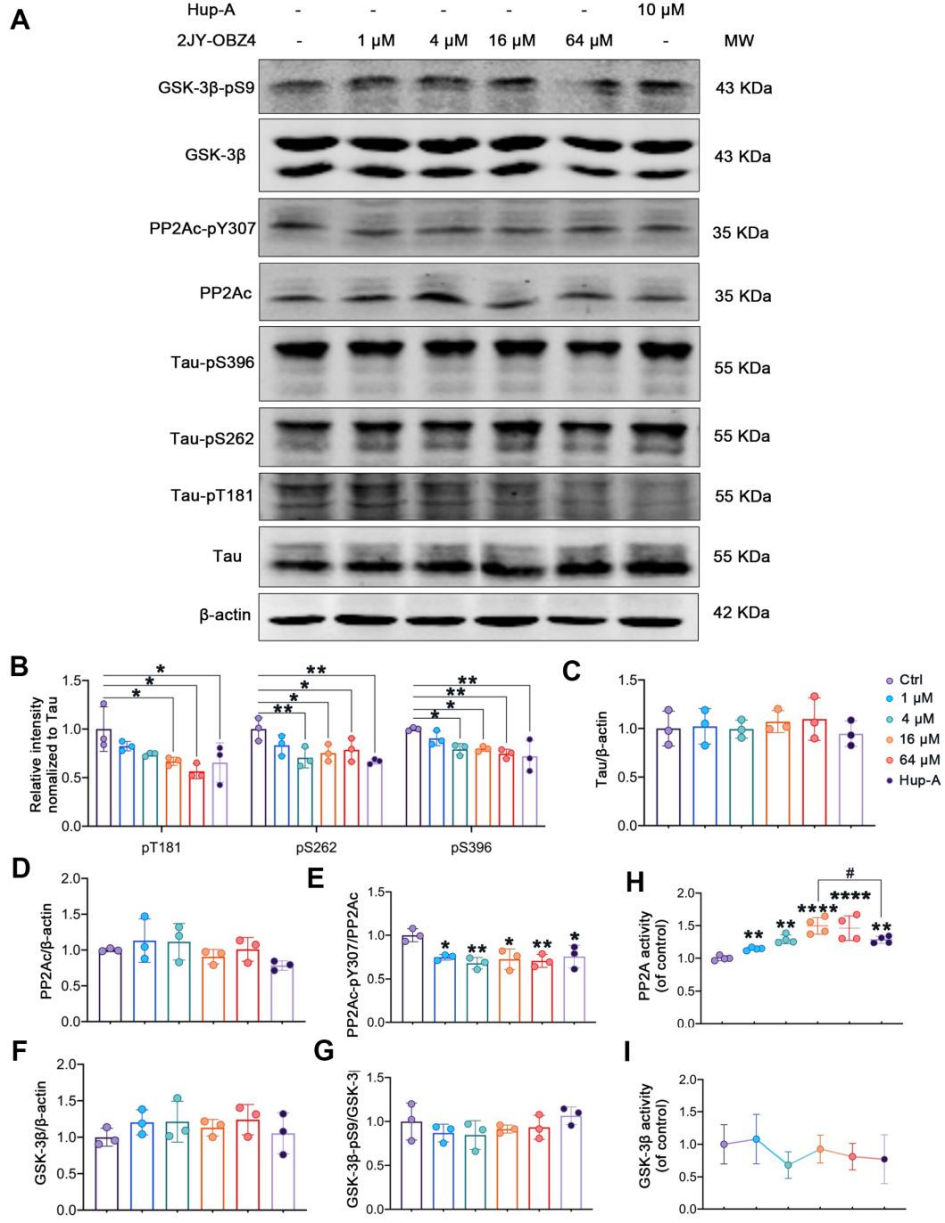
**2JY-OBZ4 induced tau dephosphorylation in HEK293/tau cells via upregulating the activity of PP2A.** (**A**) Western blots and (**B**–**G**) quantitative analysis for Tau-pT181, Tau-pS262, Tau-pS396, Tau, PP2Ac, PP2Ac-pY307 (PP2Ac phosphor-Tyr^307^, an indicator of inhibition of PP2A), GSK-3β, GSK-3β-pS9 (GSK-3β phosphor-Ser^9^, an indicator of inhibition of GSK-3β) in HEK293-hTau cells. PP2Ac represents catalytic subunit of PP2A holoenzyme. MW Molecular weight. n = 3 per group. (**H**) PP2A activity was detected in HEK293-hTau cells. n = 4 per group. (**I**) GSK-3β activity was detected in HEK293-hTau cells. n = 3 per group. p value significance is calculated from a one-way ANOVA, data are represented as mean ± SEM. *p < 0.05, ** p < 0.01, **** p < 0.0001, compared to controls and # p < 0.05.

To evaluate the direct effect of 2JY-OBZ4 on tau phosphorylation, HEK293-hTau cells were incubated with different concentrations of 2JY-OBZ4 for 24 h. 10 μM Hup-A was used as positive control. Western blot results showed that 16 μM and 64 μM 2JY-OBZ4 reduced tau phosphorylation level of Thr181 site; 4 μM, 16 μM and 64 μM 2JY-OBZ4 reduced tau phosphorylation level of Ser262 site; 4 μM, 16 μM and 64 μM 2JY-OBZ4 reduced tau phosphorylation level of Ser396 site (Figure 3A, 3B). Meanwhile, Hup-A decreased tau phosphorylation level of all the three sites (Figure 3A, 3B). Total tau had no change (Figure 3A, 3C). It is noticed that 2JY-OBZ4 had comparable effects on tau dephosphorylation to that of Hup-A (Figure 3A, 3B).

Two classes of enzymes, protein kinases and protein phosphatases, affect phosphorylation state of Tau. To be specific, protein kinases add a phosphate group (PO43−) to specified sites of tau and protein phosphatases remove these groups [19]. Phosphatases of tau include PP2A, PP2B, PP1 and PP5 and so on, of which, PP2A is particularly the main phosphatase and responsible for more than 70% of Tau Ser/Thr dephosphorylation in human brain [20]. Tau kinases include glycogen GSK-3β, MAP kinase (MEK1/2), neuronal cdc2-like kinase (NCLK) and cyclin dependent kinase 5 (cdk5) and so on, of which, GSK-3β plays a crucial role in tau phosphorylation [18]. Maintaining the dynamic equilibrium between the opposing activities of kinases and phosphatases is responsible for the regulation of phosphor-tau level. As we know, phosphorylation of an enzyme is a way to regulate its activity. For instance, phosphorylation at PP2Ac Tyr307 is associated with PP2A’s inhibitory activity [21, 22], and phosphorylation at Ser9 lead to inactivity of GSK-3β [23, 24]. Hence, we measured the protein levels of tau, PP2Ac, GSK-3β and their phosphorylation by western blot. We observed that PP2Ac phosphorylated at Tyr307 was decreased after treatment with various concentrations of 2JY-OBZ4 in HEK293-hTau cells and no change in total PP2Ac level (Figure 3A, 3D, 3E). As positive control, Hup-A also reduced PP2Ac phosphor-Tyr307 level in HEK293-hTau cells (Figure 3A, 3D, 3E). To further verify the effects of the compounds on PP2A activity, PP2A activity was detected by Serine/Threonine Phosphatase Assay kit. It turned out that 4 μM, 16 μM and 64 μM 2JY-OBZ4 induced the activation of PP2A, and Hup-A also increased the activity of PP2A (Figure 3H). Moreover, it was observed that 16 μM 2JY-OBZ4 had more potent effects on PP2A activity than Hup-A (Figure 3H, p < 0.05). There was no difference in the levels of GSK-3β and GSK-3β phosphor-Ser9 among all groups (Figure 3A, 3F, 3G). Activity of GSK-3β was detected by ELISA, and it turned out 2JY-OBZ4 had no effects on GSK-3β activity in HEK293-hTau cells (Figure 3I). These results suggested that 2JY-OBZ4 could decrease tau phosphorylation level in HEK293/tau cells via upregulating the activity of PP2A.

### 2JY-OBZ4 reduced Aβ level via regulating the levels of APP cleavage enzymes in N2a-hAPP cells

**Figure 4:**
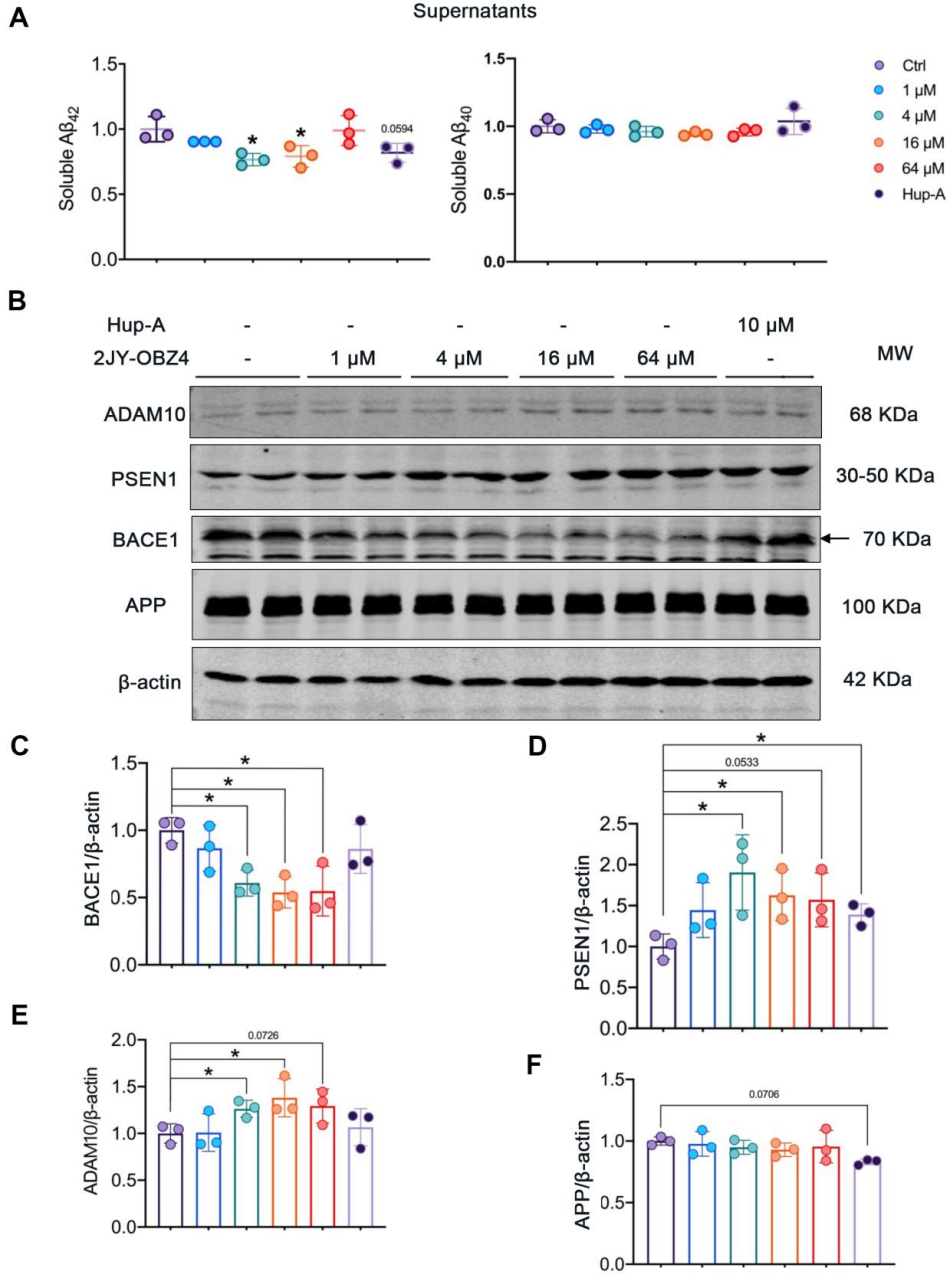
**2JY-OBZ4 reduced Aβ level via regulating the levels of APP secretases in N2a-hAPP cells.** (**A**) ELISA assessment of soluble Aβ_42_ and Aβ_40_ in the supernatants of N2a-hAPP cells after treatment with DMSO, 2JY-OBZ4 or Hup-A for 24 h. (**B**) Western blots and (**C**–**F**) quantitative analysis for BACE1, PSEN1, ADAM10 and APP in N2a-hAPP cells. PSEN1 represents catalytic subunit of γ-secretase holoenzyme. MW Molecular weight. n = 3 per group. p value significance is calculated from a one-way ANOVA, data are represented as mean ± SEM. *p < 0.05, compared to controls.

Aβ plaque is one of neuropathological hallmarks in AD brains, and turns to be an important drug target for AD [3]. In our previous study, 2JY-OBZ4 could reduce the secretion of Aβ42 in supernatants of N2a-hAPP cells (with stable hAPP expression) (unpublished data). In this work, firstly we wanted to explore the effects of different concentrations of 2JY-OBZ4 on Aβ production. Hence, N2a-hAPP cells were treated with 1 μM, 4 μM, 16 μM and 64 μM 2JY-OBZ4 for 24 h, with 10 μM Hup-A as positive control [12], thereby soluble Aβ42 and Aβ40 in supernatants of N2a-hAPP cells were detected by ELISA. Aβ42 was decreased when treated with 4 μM and 16 μM 2JY-OBZ4, nevertheless, there is no significant difference between Hup-A group and control group (Figure 4A), suggesting that 2JY-OBZ4 had better effects on decreasing Aβ production.

Next, we wanted to further illustrate the underlying mechanisms by which 2JY-OBZ4 affects Aβ production. Aβ is generated proteolytically from amyloid precursor protein (APP) by a group of secretases, including α-secretase, β-secretase (BACE1) and γ-secretase [25]. Toxic Aβ segments start with BACE1 cleavage of APP, thereby APP turns into APP secreted β fragment (sAPPβ) and C-terminal fragment (C99). C-terminal fragment can be subsequentially cleaved by γ-secretase, and forms Aβ peptide and a cell-membrane-bound fragment [26]. That is the pathway to produce Aβ, named APP amyloidogenic pathway (Figure 4D). However, APP can also be cleaved by α-secretase to produce APP secreted α segment (sAPPα) and C83, which prevents Aβ production [26]. The process is called APP non-amyloidogenic pathway (Figure 4D). Among the three proteases, BACE1 is required for the generation of all forms of monomeric Aβ peptide, therefore it has been thought to play a crucial role in AD pathophysiology [27]. Hence, gradient concentrations of 2JY-OBZ4 were treated in N2a-hAPP cells for 24 h, with Hup-A as positive control. Afterwards, protein levels of BACE1, PSEN1 (catalytic subunit of γ-secretase complex) and ADAM10 (a major α secretase) were detected by western blot. As rate-limiting enzyme of Aβ production, BACE1 was decreased when treated with 4 μM, 16 μM and 64 μM 2JY-OBZ4 (Figure 4B, 4C). However, PSEN1, another enzyme cleaving APP to produce Aβ, was increased when treated with various concentrations of 2JY-OBZ4 (Figure 4B, 4D). In our previous research, sAPPα was increased after treatment with 2JY-OBZ4 (unpublished data), indicating α-secretase was upregulated. In this study, it was confirmed that ADAM10, a major α secretase, was increased when treated with various concentrations of 2JY-OBZ4 (Figure 4B, 4E). All together, these results suggested that after treatment with 2JY-OBZ4, more APP was cleaved by α-secretase and γ-secretase, to produce sAPPα, on the other side, less APP was cleaved by BACE1, thereby less Aβ peptide was produced then. Hup-A had no effects on BACE1 and ADAM10, but increased the level of PSEN1, in addition, induced a little bit decrease in APP level (Figure 4B–4F).

### 2JY-OBZ4 ameliorated human oligomeric Aβ42-induced synaptic loss in mice primary neuron

**Figure 5:**
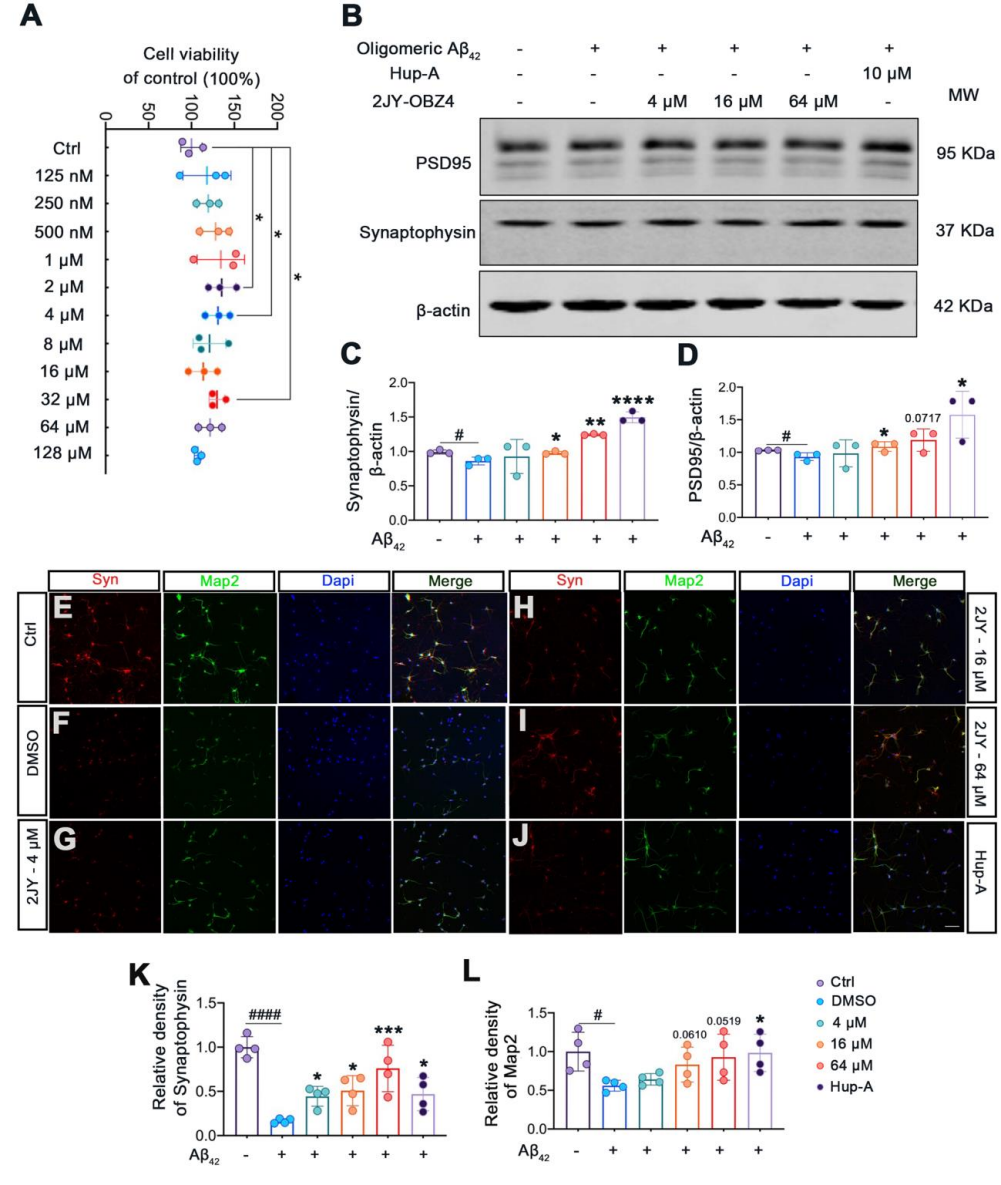
**2JY-OBZ4 ameliorated human oligomeric Aβ42 induced synaptic loss in mice primary neuron.** (**A**) CCK-8 assay of mice primary neuron incubated with various concentrations of 2JY-OBZ4 (0 nM, 125 nM, 250 nM, 500 nM, 1μM, 2 μM, 4 μM, 8 μM, 16 μM, 32 μM, 64 μM, 128 μM) for 24 h. n = 3 per group. p value significance is calculated from a one-way ANOVA, data are represented as mean ± SEM. *p < 0.05, compared to controls. (**B**) Western blots and (**C**, **D**) quantitative analysis for synaptophysin and PSD95 in mice primary neuron. MW Molecular weight. n = 3 per group. #p < 0.05 compared to controls, *p < 0.01, **p < 0.01, ****p < 0.0001 compared to the group pretreated with Aβ and treated with DMSO (**E**–**J**) Immunofluorescence staining was used to measure the expression of synaptophysin and Map2 in primary neuron (scale bar: 50 μm). (**K**, **L**) Quantitative analysis of fluorescence intensity. n = 4 per group. p value significance is calculated from a one-way ANOVA, data are represented as mean ± SEM. #p < 0.05, #### p < 0.0001 compared to controls, *p < 0.01, ***p< 0.001 compared to the group pretreated with Aβ and treated with DMSO.

Firstly, to evaluate whether 2JY-OBZ4 had adverse effects on mice primary neuron, we detected cell viability of mice primary neuron treated with various concentrations of 2JY-OBZ4 via CCK-8 assay. The results showed that various concentrations of 2JY-OBZ4 had no adverse on cell viability of mice primary neuron, in particular, 2 μM, 4 μM and 32 μM 2JY-OBZ4 significantly increased cell viability (Figure 5A).

Numerous evidence suggest that soluble Aβ oligomers induce synaptic loss in AD [28]. Soluble Aβ oligomers directly isolated from cerebral cortex of AD patients have been shown to potently impair synapse structure and function [29]. We therefore next explored whether 2JY-OBZ4 treatment has a potential effect to ameliorate synaptic loss induced by human oligomeric Aβ42 in mice primary neuron. The results as evaluated by western blot showed that treatments with 16 μM and 64 μM 2JY-OBZ4 increased synaptophysin and PSD-95 in mice primary neuron pretreated with human oligomeric Aβ42 (Figure 5B–5D). Moreover, immunofluorescence staining also identified increased level of synaptophysin in the group treated with various concentrations of 2JY-OBZ4 of Aβ42 pretreated neurons (Figure 5E–5I, 5K). 16 μM and 64 μM 2JY-OBZ4 also rescued Map2 loss in primary neuron pretreated with Aβ42 (Figure 5E–5I, 5L). As positive control, Hup-A had better effects on alleviating Aβ42 induced synaptic loss in primary neuron (Figure 5B–5L). These findings, therefore, suggested that high dose 2JY-OBZ4 treatment rescued the Aβ-induced toxicity in primary neuron.

### Evaluations of 2JY-OBZ4’s AChE inhibitory activity on mice primary neuron

We previously had discovered that 10 μM 2JY-OBZ4 could decrease AChE activity for 17% in primary neuron (unpublished data). To further quantification the potential activity of 2JY-OBZ4 on inhibiting AChE, gradient concentrations of 2JY-OBZ4 (2 μg/ml, 4 μg/ml, 8 μg/ml, 16 μg/ml and 32 μg/ml) were treated in mice primary neuron, thereby AChE of primary neuron was detected by Acetylcholinesterase Assay Kit, and lastly half maximal inhibitory concentration (IC50) was calculated. A low IC50 value suggests better efficacy and lower potency of drug required for enzyme inhibition. Hup-A, a well-known AChE inhibitor, was utilized as positive control. 2JY-OBZ4 showed weaker AChE inhibitory activity compared to Hup-A, with IC50 to be 12.35 μg/ml (Table 1). 8 μg/ml Hup-A exhibited comparable enzymatic inhibitory effect to 32 μg/ml 2JY-OBZ4 (Table 1). Despite the fact that 2JY-OBZ4 was weaker on AChE inhibitory activity than Hup-A, in the meanwhile, it indicated that the cholinergic side effects of 2JY-OBZ4 would be much weaker than Hup-A.

**Table 1:** Percentage of AChE inhibition and IC_50_ results for 2JY-OBZ4.

**Test compounds**	**% Inhibition**					**IC_50_ (μg/ml)**	**95% Confidence interval of IC_50_ (μg/ml)**
	**2μg/ml**	**4 μg/ml**	**8 μg/ml**	**16 μg/ml**	**32 μg/ml**		
2JY-OBZ4	14.23	23.74	30.70	39.14	47.11	39.48	35.53, 44.60
Huperzine A	37.10	41.29	47.50	52.02	56.60	12.35	11.03, 13.95

Half maximal inhibitory concentration (IC_50_) and 95% confidence interval of IC_50_ was calculated by nonlinear regression. Huperzine A, Hup-A.

## DISCUSSION

AD is associated with the onset of significant and progressive disability throughout the disease course, with death an inevitable outcome generally occurring within 5–12 years of symptom onset [30]. There is desirable need for therapies that could prevent or slow down the progression of AD. Unfortunately, pharmaceutical development for AD, most of which aimed at slowing down the progression of AD, has come with endless failures in mid-to-late-stage clinical trials [1]. Though the therapeutic exploration for AD is confronted with an enormous challenge, and some pharmaceutical companies even choose to abandon their AD drug development divisions, researchers all over the world never stop developing and testing novel therapeutics for AD.

AD always makes its progression from preclinical disease, to mild cognitive and/or behavioral impairment and then Alzheimer's disease dementia [31]. By the time it is typically diagnosed, substantial neuronal loss and neuropathological lesions have already damaged many brain regions, causing great difficulties for the treatment [32]. Hence, pharmaceutical intervention at early stage of AD may be a direction of future anti-AD drugs developments. In recent years, early diagnosis of AD by molecular markers in peripheral blood are under research. Plasma tau phosphorylated at threonine 181 (p-tau181) was found to be increased in preclinical AD and further increased at the MCI and dementia stages, making a noninvasive diagnostic and prognostic biomarker of AD [33]. Plasma tau phosphorylated at threonine 217 (p-tau217) was also proved to differentiate diagnosis of Alzheimer's disease syndromes from frontotemporal lobar degeneration (FTLD) syndromes, as an indication of amyloid-PET-positivity, and to have stronger correlations with tau-PET signal [34].

Studies of AD pharmaceutical intervention are mainly focused on cholinesterase inhibition, anti-NMDA, Aβ-directed therapeutics, tau-directed therapeutics, ApoE-directed therapeutics and combination therapies [1]. Combination therapies mean therapies targeted at more than one pathological characteristic, which is suitable for AD prevention in ideal conditions. Novel small molecular compound 2JY-OBZ4 was found to act on multiple AD related pathological phenomena, such as tau hyperphosphorylation, Aβ toxicity and decreased acetylcholine (ACh), making it a member of combination therapies.

Extracellular amyloid plaque is a pathological hallmark of AD, with Aβ as its major component [3, 35]. Compared to Aβ40, Aβ42 is more prone to aggregation, owning to its increased hydrophobicity of expanded C terminus [36, 37]. Aβ is produced predominantly in endosomes, generating from cleavage of APP by BACE1 and γ-secretase [38]. The γ-secretase complex consists of four protein subunits: presenilin (PSEN), presenilin enhancer (PEN), APH, and Nicastrin. There are multiple isoforms of PSEN (PSEN1/PSEN2) and APH (APHA, APH B/C) [39]. Thereinto, PSEN1 is the primary catalytic subunit of γ-secretase complex. A PSEN1 mutation that causes a nearly complete loss of γ-secretase activity was found in familial AD cases [40], which supports the perspective that PSEN1 plays a crucial role in the normal activity of γ-secretase complex. BACE1, an aspartyl protease providing first cleavage in APP, is thought to be the rate-limiting enzyme for Aβ production. BACE1 inhibitor could reduce Aβ generation in AD mice brains and AD patients’ brains [41]. In particular, both BACE1 and PSEN1 are found within amyloid plaque of human AD brain [35]. In our research, increased PSEN1 and decreased BACE1 were observed in N2a-hAPP cells treated with 2JY-OBZ4. As a result, Aβ42, which has a greater propensity for aggregation than other Aβ peptides, was found to be reduced in cell supernatants.

On the other hand, Aβ is toxic to neurons in a myriad of ways. When neurons release Aβ to the extracellular environment, it would drive other AD pathologies like tau pathology, dendritic and synaptic dysfunction [28, 29, 42, 43]. Here, western blot and immunofluorescence showed that synaptic proteins as synaptophysin and PSD95 were decreased after treatment with human oligomeric Aβ42 in rodent neurons in accordance with findings in previous studies [28, 44–46]. Synaptophysin is the major integral membrane protein of synaptic vesicles, functioning in synaptic vesicle cycling, endocytosis, and synaptic plasticity [47], which is always used as synaptic marker to obtain the reliable data on the morphological organization of the synaptic structures in neurons with the use of confocal laser microscopy. PSD95 is an important postsynaptic scaffolding protein which is involved in recruiting and anchoring glutamate receptor subunits to the post-synaptic density [48]. However, 2JY-OBZ4 attenuated oligomeric Aβ induced synaptophysin and PSD95 protein loss in the current study. Map2, one of major cytoskeletal components in neurons, has been proposed to play critical roles in the stabilization of microtubules, the regulation of organelle transport within axons and dendrites, the outgrowth of neuronal processes, the nucleation and so on [49]. In the present study, Map2 was impaired with pretreatment of Aβ oligomers, afterwards, the effects were reversed by treatment of 2JY-OBZ4. In all, 2JY-OBZ4 alleviated synaptic structural damages induced by Aβ oligomers in rodent primary neuron. Nevertheless, the underlying mechanisms requires further clarity.

Intraneuronal neurofibrillary tangle formed by hyperphosphorylated tau is another pathological hallmark of AD [3]. The burden of cortical neurofibrillary tangles correlates better with the progression of cognitive impairment than Aβ plaques in the brains of AD patients [50]. Tau protein is encoded by MAPT gene on chromosome 17 of human genome, as a major microtubule-associated protein in neurons, bearing physiological roles in microtubule assembly, stabilization of neuronal axons, and regulation of microtubule transport [51]. Abnormal post-translational modifications (PTMs) of tau, especially hyperphosphorylation, lead to tau aggregation and fibrillization [52]. In addition to this, hyperphosphorylated tau could spread from the axonal compartment to dendritic spines where it impairs synaptic function by inhibiting glutamate receptor trafficking or synaptic anchoring [53]. During the recent years, it has been demonstrated that aggregated tau has prion-like ability, to spread one specific brain region to anatomically connected brain regions, inducing further seeding and aggregation [54, 55]. It can be inferred from above evidence that tau pathology plays an important role in AD pathogenesis. Therapeutics to reduce tau pathologies are primarily focused on tau aggregation inhibition [56] and abnormal hyperphosphorylation inhibition [57]. In our current study, 2JY-OBZ4 was found to decrease phosphorylation level of tau at various sites by means of impacting the activity of tau phosphatase, PP2A.

PP2A is a major serine/threonine phosphatase which participates in the regulation of multiple cellular processes such as metabolism, neural development, transcription, cell cycle, proliferation, and apoptosis [58]. As a confirmed tumor suppressor, PP2A activity is downregulated in tumors and its reactivation can induce apoptosis of cancer cells [59]. Compromised PP2A activity is also observed in the brains of AD patients [60, 61]. The majority of PP2A holoenzyme are heterotrimers, consisting of one catalytic C subunit, one structural A subunit, and one variable regulatory B-type subunit [62]. An important regulatory portion of PP2Ac subunit, the catalytic subunit, is its conserved C-terminal tail (Thr304-Pro-Asp-Tyr-Phe-Leu309). A number of biochemical and structural studies have underscored that post translational modifications of this tail contribute to or interfere with holoenzyme assembly, thereby impact the enzymatic activity [62]. Phosphorylation of PP2Ac at Tyr307 and Thr304 sites, which is catalyzed by a cyclin-dependent kinase (CDK) and a Src-like or receptor tyrosine kinase (RTK), plays an inhibitory role in the regulation of PP2A activity [58]. Another important post-translational modification of PP2Ac is reversible methylation of C-terminal Leu309, which is catalyzed by leucine carboxyl methyl transferase 1 (LCMT1) and reversed by PP2A methyl esterase 1 (PME-1) [63]. Methylation at Leu309 was found to be an absolute prerequisite for recruitment of PR55/B subunit, a B-type subunit [62]. In our study, the phosphorylation level of PP2Ac-Tyr307 was decreased when treated with 2JY-OBZ4. It can be suggested that the compromised phosphorylation modification at Tyr307 lessened its inhibition to the holoenzyme assembly, thereby, PP2A activity was increased; the deeper mechanism by what means 2JY-OBZ4 decreased the phosphorylation at Tyr307 remains to be established.

Except for tau phosphatase, tau kinase is another determinant for tau phosphorylation. Since GSK-3β is responsible for tau phosphorylation at Thr181 site [18], we detected the activity of GSK-3β by GSK-3β activity assay kit. The results showed that the activity of GSK-3β didn’t change. However, other tau kinases like SAPK, DYRK1A, JNK, ERK1/2 and CDK5 also contribute to the phosphorylation of Thr181 site [18]. Whether 2JY-OBZ4 decreased the phosphorylation of tau Thr 181 site by regulating the activity of these kinases needs further exploration.

Hup-A was approved to treatment of mild to moderate AD because of its inhibitory activity on AChE [13]. Owing to its common structure with Hup-A, 2JY-OBZ4 showed moderate AChE inhibitory activity, which was weaker than Hup-A. From the available evidence, cholinesterase inhibitors like donepezil, galantamine, rivastigmine and Hup-A, showed symptomatic benefits in AD that could improve cognitive performance and global functioning [13, 64], though the brain damage was not terminated [65]. These lines of evidence proves that the AChE activity of 2JY-OBZ4 is still beneficial in AD therapy.

In summary, 2JY-OBZ4 showed promising therapeutic effects in AD cell models through regulating multiple targets including PP2A activity, APP cleavage enzymes and AChE activity. Compared to Hup-A, 2JY-OBZ4 had moderate AChE inhibitory activity with two times higher IC50, supporting that 2JY-OBZ4 had a wider drug safety window. In our study, high dose 2JY-OBZ4 had more potent effects on resisting tau hyperphosphorylation in HEK293-hTau cells overexpressed with GSK-3β, activating PP2A, decreasing Aβ production and ameliorating synaptic loss induced by Aβ42. The research provides a new candidate for the therapeutic development of Alzheimer’s disease based on multi-target strategy.

## MATERIALS AND METHODS

### Chemicals and antibodies

2JY-OBZ4 was synthesized chemically by the lab of Prof. Yang Yang (School of Pharmacy, Huazhong University of Science and Technology, China), and the molecular formula of 2JY-OBZ4 was confirmed by 2D NMR experiments (COSY, HSQC, HMBC, and NOESY) [14]. Huperzine A (purity: 98%) was purchased from MedChemExpress, USA.

The primary antibodies utilized in this study are as follows: GSK-3β (21002, Signalway Antibody), tau (R25863, Zen-Bioscience), tau-pT181 (R23342, Zen-Bioscience), tau-pS396 (381213, Zen-Bioscience), tau-pS262 (310195, Zen-Bioscience), PP2Ac (R25422, Zen-Bioscience), PP2A-pY307 (380708, Zen-Bioscience), GSK-3β-pS9 (5558, Cell Signaling Technology), APP (R22718, Zen-Bioscience), ADAM10 (A10438, Abclonal), BACE1 (A11533, Abclonal), PSEN1 (A19103, Abclonal), Synaptophysin (sc-17750, Santa Cruz Biotechnology), PSD95 (MABN68, Merck Millipore), Map2 (ab254264, Abcam), β-actin (AC026, Abclonal). The secondary antibodies include Goat anti-Rabbit IgG (Li-Cor Biosciences), Goat anti-Mouse IgG (Li-Cor Biosciences), Goat anti-Rabbit IgG Alexa Fluor Plus 488 (Invitrogen), and Goat anti-Mouse IgG Alexa Fluor Plus 555 (Invitrogen).

### Continuous cell culture and plasmids transfection

Human embryonic kidney 293 cell line with stable expression of full-length human tau (termed as HEK293-htau) and mouse neuroblastoma cell line with stable expression of full-length human APP (termed as N2a-hAPP) were cultured in a humidified incubator aerated with 5% CO2 at 37° C. Cells were cultured in DMEM (Gibco, USA) media supplemented with 10% fetal bovine serum (FBS, Gibco, USA) and antibiotics (100 U/mL penicillin and 100 μg/mL streptomycin). For western blot, cells were seeded into 6-well plates and cultured until the cell density reached 80%. Then changed the medium with DMEM dissolved with 2JY-OBZ4 or Hup-A or the same volume of DMSO. After incubating for 24 h, the cell protein extract was collected.

HEK293-hTau cells were seeded into 6-well plates at least 24 h before transfection with Neofect DNA transfection reagent (Neofect Biotech Corporation, China). When the cell density reached 60%-80%, it’s appropriate to do the transfection following the manufacturer’s instruction. After 36-48 h, when plasmid expression reached its peak, HEK293-hTau cells were treated with 2JY-OBZ4 or Hup-A or DMSO. The plasmid pCDNA3.0-GSK-3β WT was a generous gift from Dr. Zhou Qiuzhi (Hubei Key Laboratory of Education Ministry of China, Wuhan, China) [66].

### Primary neuron culture and treatment

Mice primary neurons were isolated from embryonic day E17 to E18 C57 mice and cultured as previous described [67]. Neurons were cultured in neurobasal medium supplemented with B27, GlutaMAX and antibiotics. Neurons were cultured for 8 days before treatments and the medium was half-changed every 3 days with fresh maintenance media during the culture. On day 9, the neurons were pretreated with 2 μM oligomeric Aβ42 for 48 h, then the compound to be tested or the same volume of DMSO were diluted in maintenance medium and respectively treated to the neurons. After 24 h, the neuron protein extract was collected. The AChE activity was checked with Acetylcholinesterase assay kit on day 9 after the neurons were treated with 2JY-OBZ4, Hup-A or DMSO for 24 h. All reagents needed for primary neuron culture were purchased from Gibco, USA.

### Reagent preparation

Recombinant Aβ42 peptide (China Peptides, China) was originally lyophilized powder. The powder was firstly dissolved in DMSO and diluted in complete medium of primary neuron to 200 μM. Then the diluted Aβ was incubated at 4° C for 14-16 h for oligomeric formation. Before treated on neurons, the oligomeric Aβ was sonicated for 30 s and diluted in cell medium to a final concentration of 2 μM. Hup-A powder (HY-17387, MCE) was dissolved in DMSO to 40 mM, and diluted in DMEM or complete medium of primary neuron to final concentration of 10 μM before use. 2JY-OBZ4 powder was dissolved in DMSO to 40 mM, and diluted in DMEM or complete medium of primary neuron to final concentrations of 1 μM, 4μM, 16 μM and 64 μM.

### CCK-8 cell viability assay

The viability of mice primary neuron treated with 2JY-OBZ4 was determined by the CCK-8 cell viability assay kit (Biosharp, China, BS350B). Neurons were seeded at a concentration of 5,000 per well in a 96-well plate, when cultured to Day 8, the neurons were treated with various concentration of 2JY-OBZ4 (0, 125 nM, 250 nM, 500 nM, 1 μM, 2μM, 4μM, 8 μM, 16 μM, 32 μM, 64 μM, 128 μM) for 24 h. After the treatments, the medium was removed, and 10 μl of CCK-8 reagent in 100 μl of medium was added. After incubating for 30 min at 37° C, the absorbance was measured using microplate reader (BioTek, 250058) at 450 nm.

### Western blot

Cells were washed twice with cold PBS. Then the cells were homogenized in RIPA buffer and 4×buffer containing PMSF (Thermo Scientific, 36978) and cocktail (MCE, HY-K0010). The homogenates were boiled for 10 min and then centrifuged (12000×g, 15 min, 4° C). The supernatants were collected and the protein concentration was assessed by BCA Protein Assay Kit (Beyotime, P0011). If fresh proteins weren’t used immediately, they were stored in - 80° C.

Cell proteins were boiled for 5 min after taking from - 80° C. The proteins were loaded in 10% SDS-polyacrylamide gel. We used 5 μg proteins to detect the level of β-actin, 10 μg for GSK-3β, tau-pT181, tau-pS396, tau, tau-pS262, PP2Ac, PP2Ac-pY307, GSK-3β-pS9, APP, PSEN1, ADAM10, synaptophysin, PSD95 and 15 μg for BACE1. Then the proteins were electrophoresed for 1-1.5 h. Then the proteins were transferred to nitrocellulose membranes (Amersham Biosciences). After blocking with 5% non-fat milk dissolved in TBS at room temperature for 1 h, the membranes were incubated in primary antibody overnight at 4° C. Then the proteins were incubated with secondary antibodies at room temperature for 1 h and visualized using the Odyssey Infrared Imaging System (Li-Cor Bioscience, USA). Image J software (Rawak Software, Germany) was utilized to quantitatively analyze the protein bands.

### PP2A activity assay

The phosphatase kit V2460 was used to measure PP2A activity in the cell extracts according to the manufacturer’s procedure (Promega, USA). In brief, supernatants of HEK293-hTau cells were prepared. Then the endogenous free phosphate was removed from supernatants by passing Sephadex G-25 retin spin columns. After assessing protein density by BCA Protein Assay Kit (Beyotime, P0011), 5 μg protein samples in double were incubated with a chemically synthesized phosphor-peptide, an optimal substrate for PP2A, PP2B, and PP2C, but not for PP-1 in a buffer optimized for PP2A activity, while cation-dependent PP2B and PP2C were inhibited. Phosphate release from the substrate was detected by measuring the absorbance of a molybdate-malachite green-phosphate complex at 630 nm. The activity of PP2A was evaluated by the release of phosphate per μg protein (pmol/ μg).

### GSK-3β activity assay

The GSK-3β assay kit was utilized to measure GSK-3β activity in the cell extracts according to the manufacturer’s procedure (JKBio, China). Human glycogen synthase kinase 3β (GSK3β) level was determined by enzyme-linked immunosorbent method. The microporous plate was pre-coated with the antibody of GSK-3β-pT216 (GSK-3β phosphorylated at Thr216 site), which positively regulates the activity of GSK-3β. The absorbance was measured using micro-plate reader at 450 nm.

### ELISA quantification of Aβ40 and Aβ42

The concentrations of Aβ40 and Aβ42 in the culture supernatants of N2a-hAPP cells were quantified using the Human Aβ1-40 or Aβ1-42 ELISA Kit (Elabscience, China) according to the manufacturer’s instructions. In brief, cell culture supernatants were collected and centrifuged at 2000 g for 20 min, then the supernatant was collected and added to the provided ELISA plate pre-coated with the anti-Aβ antibody. After incubating for 90 min at 37° C, liquid in the plate was discarded and washed. The plate wells were incubated with biotinylated detection antibody working solution for 1 h at 37° C. Then liquid in the plate was discarded and washed again. The plate wells were incubated with HRP conjugate working solution for 30 min, washed, and incubated with a substrate reagent for 15 min at 37° C. At last, the stop solution was added and the optical density of detected wells were measured using micro-plate reader at 450 nm.

### Immunofluorescence

Cultured neurons were fixed in 4% paraformaldehyde (vol/vol) for 30 min at room temperature and permeabilized in 0.5% Triton X-100 (vol/vol) diluted in PBS solution. Nonspecific binding sites were blocked via incubating in 5% (wt/vol) BSA containing 0.1% Triton X-100 (vol/vol) for 30 min. The samples were incubated with anti-Synaptophysin (sc-17750, Santa Cruz Biotechnology) dilution at 4° C overnight, followed by washing 3 times in PBS and subsequent incubation with anti-Map2 (ab254264, Abcam) dilution. Then the samples were washed 3 times in PBS and subsequent incubated with mixed secondary antibodies (Goat anti-Rabbit IgG Alexa Fluor Plus 488 and Goat anti-Mouse IgG Alexa Fluor Plus 555) diluted in PBS solution for 1 h at 37° C. After washing 3 times in PBS, the samples were further stained with anti-fluorescence quenching sealing liquid containing Dapi (Beyotime, China) and finally mounted onto slides. All the slides were imaged with a confocal microscope (Zeiss Carl LSM 780, Germany).

### Acetylcholinesterase activity assay

The acetylcholinesterase (AChE) activity of primary neurons lysates was carried out following the protocol in the Acetylcholinesterase assay kit (Abcam, ab138871). This kit detects AChE activity using DTNB to quantify the thiocholine produced from hydrolysis of acetylthiocholine by AChE. The absorption intensity of DTNB adduct (410 nm) is used to measure the amount of thiocholine formed, which is proportional to the AChE activity. The assay can detect as little as 0.1 mU AChE in a 100 μL assay volume (1 mU/mL), which served as a reliable and sensitive test for this study. In brief, Neurons were cultured for 9 days before respectively treatment with gradient concentrations of 2JY-OBZ4 or Hup-A (2 μg/ml, 4 μg/ml, 8 μg/ml, 16 μg/ml and 32 μg/ml). After 24 h, cells were collected with RIPA lysis buffer. Then 50 μl cell lysates samples (test samples) were added to a 96-well plate; 50 μl assay buffer was used as blank control; 50 μl serial dilutions of standard acetylcholinesterase were utilized to construct standard curve. Acetylthiocholine-reaction mixture was prepared and 50 μl of which was added to each well of the test samples, blank control, and the acetylcholinesterase standard. After incubation for 10 to 30 mins at room temperature under the dark condition, the each well absorbance was measured with microplate reader at 410 nm. The protein concentration of test samples was assessed by BCA Protein Assay Kit, then the protein value of each sample well was calculated. The sample well absorbances were compared to the standard curve values and the AChE amounts (mU) were normalized to protein values (mU/ mg protein).

### Statistical

All data were expressed as mean ± SEM and analyzed using Graph Pad Prism 8 software (San Diego, CA, USA). Difference between groups were assessed using one-way ANOVA, or student’s t-test. In all cases, a value of P < 0.05 was considered statistically significant.

### Availability of data and materials

The datasets used and/or analyzed during the present study are available from the corresponding author on reasonable request.
